# The parental perspective of thalassaemia in Bangladesh: lack of knowledge, regret, and barriers

**DOI:** 10.1186/s13023-021-01947-6

**Published:** 2021-07-16

**Authors:** Mohammad Sorowar Hossain, Md. Mahbub Hasan, Mary Petrou, Paul Telfer, Abdullah Al Mosabbir

**Affiliations:** 1 Department of Emerging and Neglected Diseases, Biomedical Research Foundation, Dhaka, Bangladesh; 2 School of Environment and Life Sciences, Independent University, Bangladesh, Dhaka, Bangladesh; 3 Department of Genetic Engineering and Biotechnology, University of Chittagong, Chattogram, 4331 Bangladesh; 4 Institute of Women’s Health , University College London, London, UK; 5 Centre for Genomics and Child Health, Blizard Institute, Barts and The London School of Medicine, Queen Mary University of London, London, UK

**Keywords:** Thalassaemia, Bangladesh, Prenatal diagnosis, Premarital, Parental perspective, Thalassaemia awareness

## Abstract

**Background:**

Thalassaemia, a hereditary haemoglobin disorder, is a major public health concern in some parts of the world. Although Bangladesh is in the world’s thalassaemia belt, the information on this disease is scarce. Additionally, the awareness of this life threatening, but potentially preventable disease is surprisingly poor. However, mass awareness is pivotal for the development of an effective preventive strategy. In this context, the understanding of parental perspectives is essential to grasp the magnitude of the problem. Therefore, this study aimed to investigate the parental knowledge gaps and perceptions regarding thalassemia, the barriers confronted by the parents for caring for their thalassaemic children and their attitude to prenatal screening and prenatal diagnosis.

**Methods:**

This cross-sectional study was conducted between January 2018 and December 2018 at a dedicated thalassemia hospital located in Dhaka. A structured questionnaire was used for face-to-face interviews with parents of thalassaemic children. Descriptive statistics were used to analyse data.

**Results:**

Of 365 respondents, nearly all respondents (97%) had not heard about the term, ‘thalassemia’ before the disease was diagnosed in their children; all (100%) were unscreened for carrier status prior to marriage. Mean knowledge scores were significantly higher in respondents with higher income and education. Most respondents (~ 91%) had a guilty feeling for not undergoing premarital screening. Only around 36% of them had heard about prenatal diagnosis. Approximately 25% participants would consider prenatal diagnosis in a future pregnancy, while 70% of them were unsure and only ~ 5% would decline prenatal diagnosis. Only 9.3% mothers had prenatal diagnosis in a previous pregnancy. Nearly 80% of the parents faced difficulty for obtaining blood donors regularly and a similar proportion (~ 81%) of them did not receive support from any organized blood clubs. More than 40% of the parents reported they felt socially stigmatized.

**Conclusion:**

This study suggests poor parental knowledge regarding thalassaemia including prenatal diagnosis and the challenges faced while caring for their children. These findings would be of paramount importance in planning and devising effective prevention and intervention strategies in Bangladesh as well as other countries with similar sociocultural setting.

## Introduction

Inherited haemoglobin disorders, especially thalassaemias and sickle cell anaemias, are the most prevalent monogenic diseases globally. According to the 1000 Genomes database, an estimated 14 out of 10,000 live births have a chance of acquiring haemoglobin disorders [1]. They were once considered a disease of the tropics and subtropics but are now encountered all over the world because of population migration. The World Health Organization (WHO) designated thalassaemias as a major public health concern in 2006 [2]. Every year between 300,000 and 500,000 infants are being born with a severe form of haemoglobin disorders, out of which approximately 30% have thalassaemia syndromes [2].

Thalassaemias are a group of blood disorders that originates from the inheritance of mutated globin genes resulting in decreased production of functional haemoglobin, and ultimately anaemia. A person who inherits one mutated gene is called a carrier and usually leads a completely normal life. However, those who inherit two mutated genes (one from each parent) will have the disease [3]. Patients with severe forms of this disease require regular blood transfusion and adequate iron chelation therapy to survive as there is no curative treatment for thalassaemias except bone marrow transplantation, which is only available for a limited number of patients due to the high cost and morbidity associated with the procedure [4]. The survival and quality of life of the patients with transfusion dependent thalassaemia (TDT) have been improved dramatically in high resource countries over the past few decades owing to safe transfusion practice, successful prevention programs and access to modern therapies [5]. However, the scenario is still very disappointing in the lower resource setting, where most of the children with TDT die before reaching 5 years and the average life expectancy of thalassaemia patients is around 30 years [6, 7].

Over the past few decades, the survival of transfusion-dependent thalassaemia patients improved significantly in high resource countries because of better disease-specific support programmes and accessibility to promising new therapies [5]. Unfortunately, up to 90% of patients with serious haemoglobin disorders live in developing countries with limited resources, which mainly includes sub-Saharan Africa and Asia [8]; and the prospect for these patients is grim. The burden is expected to rise substantially in the coming years in these areas due to the reduction in infant mortality and large population size. However, it is clear from many reports that most of these developing countries have extremely limited facilities for the diagnosis and management of thalassaemias, mainly due to the high cost associated with standard treatment [9]. Thus, it is not possible for resource-constrained countries to meet the ever-growing treatment demand of thalassaemic patients. Moreover, the psychological impact and social stigmatisation among patients and their caregivers is notably higher in these countries [10]. Therefore, reducing the burden of thalassaemias through effective preventive approaches in parallel with widespread awareness programmes is the most critical and cost-effective strategy for resource-poor countries.

Bangladesh is a developing country with a population of more than 165 million. Nearly 70% of its people reside in resource-limited rural areas with few facilities diagnosis and management of haemoglobin disorders [11]. Although the prevalence of thalassaemia genes in the Bangladeshi population is high, accurate data on the disease burden of thalassaemias is sparse. Conservative estimates suggest that about 6–12% of the population are carriers of different haemoglobin disorders, mainly beta-thalassaemia and haemoglobin E (HbE); the proportion reaches up to 40% in the tribal populations [12]. Extrapolated data also suggests that about 60,000–70,000 children have been suffering from clinically severe thalassaemias [11].

Detecting carriers and educating them about the disease and its outcomes is the focus of all thalassaemia prevention programmes. Common strategies include mandatory premarital screening with the option of prenatal diagnosis and therapeutic termination of pregnancy. Some countries, notably Cyprus, Greece, Iran, and Italy, have achieved high success in reducing the birth prevalence of thalassaemia by implementing these strategies [2]. Screening services and prenatal diagnosis with therapeutic termination of the affected pregnancies are also available in Bangladesh, but it is unclear how effective and accepted these preventive strategies will be in Bangladesh. In this context, effective community participation is vital, especially in developing countries like Bangladesh where overall health awareness is very poor among the general population. Thus, engaging the parents or caregivers of thalassaemic children at a community-level would be instrumental in creating mass awareness. Parents or caregivers play a vital role in chronic diseases like thalassaemias. Their knowledge and perspective regarding the disease can help the society to realise the depth and seriousness of the intertwined problems regarding the health, economic and psychological burden prevailing in families of thalassaemic children in resource-poor settings. The current study aims to investigate the parental knowledge and attitudes regarding thalassemia, prenatal diagnosis and practical barriers faced by the parents of thalassaemic children in Bangladesh.

## Materials and methods

### Study setting and participants

This cross-section study was conducted at the Bangladesh Thalassaemia Samity Hospital (BTSH) situated in the capital city, Dhaka. This was the first day-care hospital dedicated to thalassaemia care in Bangladesh, which was established in 2000. BTSH is managed by parents of thalassaemic children. There are few specialized thalassaemia care centres in Bangladesh, and most are in Dhaka city, although around 70% population live in rural areas in Bangladesh. Because of financial constraints, most patients cannot afford treatment in Dhaka. In this study, we used convenience sampling technique to recruit caregivers (mother or father) of thalassaemic children attending at BTSH for follow-up treatment (regular blood transfusion and care) in the period between January 2018 and December 2018.

The study protocol was ethically approved by the Ethical Review Committee of Bangladesh University of Health Sciences (Memo: BUHS/BIO/EA/17/100). Written informed consent was obtained from each participant.

### Instrument and data collection

For assessing general knowledge and prevention approaches regarding thalassemia, a previously published questionnaire was used [13]. The socio-demographic section of the questionnaire included the information of caregivers (such as age, sex, monthly income, education, number of thalassaemic children) and profile of thalassaemic children (including age at diagnosis, type of thalassemia, current age, and blood transfusion requirement). A multidisciplinary team of experts including a haematologist, public health researcher, parents of thalassaemia children and statistician was formed to develop another section of the study questionnaire to investigate the attitudes and practice towards prenatal diagnosis and barriers. Regarding attitude towards prenatal diagnosis, the following questions were included: have you heard of prenatal screening (yes/no)? would you accept prenatal diagnosis in a future pregnancy (yes/no/not sure)?, have you undergone prenatal diagnosis in a previous pregnancy (yes/no)? would you consider abortion after a positive prenatal test (yes/no/not sure)?. To investigate the barriers faced by parents of thalassaemic children following questions were asked: do you get support from social blood donor clubs? (yes/no), do you have difficulty in getting regular donors and social stigmatization (yes/no)? do you feel stigmatized in the society? (yes/no). The drafted questionnaire was tested on 5 parents of thalassaemic children. After that, the study questionnaire was finalised. Data collectors from the BTSH were trained conduct face-to-face interviews with parents.

### Statistical analyses

Descriptive statistical procedures were used to analyse data. Categorical variables were presented using counts and percentages, and continuous variables were summarized using means and standard deviations. Kruskal–Wallis one way ANOVA tests were performed to see the differences in knowledge scores between two groups. A *p* value < 0.05 was considered significant. Statistical analysis was done using SPSS software (version 25).

## Results

### Characteristics of participants

**Fig. 1:**
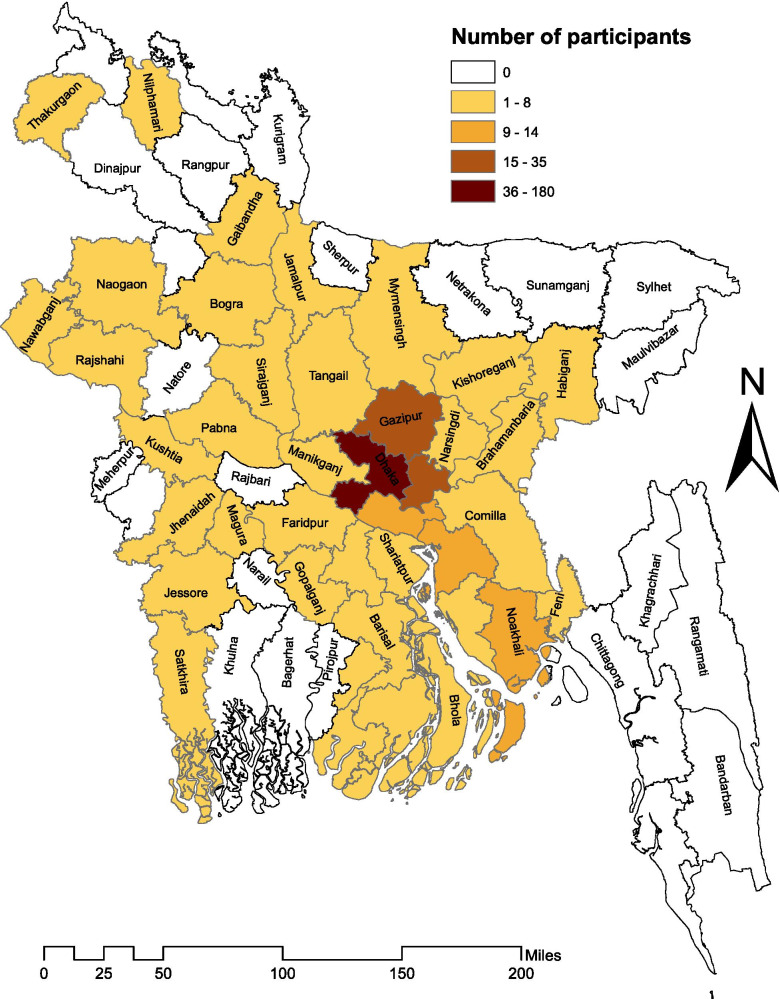
Spatial distribution of respondents across different administrative districts of Bangladesh

A total of 365 parents of children with thalassaemia from all over the country (48 administrative districts out of 64) participated in this study (Fig. 1). Of these participants, 58% were mothers. Most parents had non-consanguineous arranged marriage (~ 85%). Over half of the parents (~ 63% father and ~ 55% mother) had at least 10 years of schooling. Nearly two-thirds (~ 68%) of the parents had two or more children. Approximately 82% of the families had one thalassaemic child while 16.4% had two children with thalassemia. There were three families with three thalassaemic children each. Almost equal proportion (around 22%) of the participants had a known family history of thalassaemia and thalassaemia associated death (Table 1).

**Table 1:** Participant characteristics (parents)

Variables	n	%
Caregiver (total)	365	100
Mother	212	58.1
Father	153	41.9
*Type of marriage of parents*		
Arranged	311	85.21
Love	54	14.79
*Consanguineous marriage*		
Yes	59	16.16
No	306	83.84
*Father’s education*		
No formal education	40	11
Primary	96	26.3
Secondary	47	12.9
Higher secondary	73	20
Graduate	109	29.9
*Mother’s education*		
No formal education	42	11.5
Primary	123	33.7
Secondary	61	16.7
Higher secondary	76	20.8
Graduate	63	17.3
Monthly family income*		
< $177	132	36.2
$177–295	93	25.5
$295–590	87	23.8
> 590	53	14.5
*No. of children*		
1 child	78	21.4
2 child	153	41.9
> 2 child	134	36.7
*No. of thalassaemic child*		
1 child	302	82.7
2 child	60	16.4
> 2 child	3	0.8
*Thalassaemic patient in extended family*		
Yes	80	21.9
No	277	75.9
Don’t know	8	2.2
*Thalassaemia caused a death in extended family*		
Yes	82	22.5
No	272	74.5
Don’t know	11	3
*Heard about thalassaemia before the diagnosis in child*		
Yes	11	3
No	354	97
*Screening of thalassaemia before marriage?*		
Yes	0	0
No	365	100

*1 USD equels to 84.75 BDT

Almost all respondents (97%) in this study had not heard about the term, ‘thalassemia’ before the disease was diagnosed in their children and all (100%) were unscreened for carrier status prior to marriage (Table 1).

**Fig. 2:**
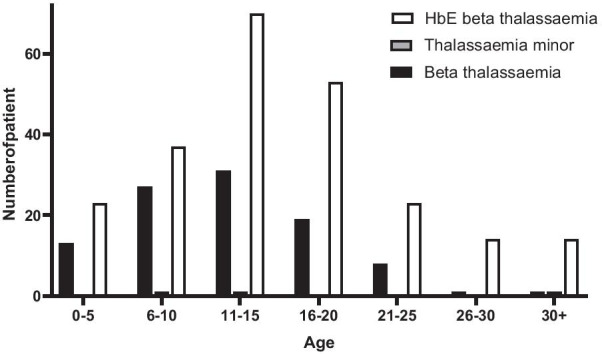
Distribution of current age of children with transfusion-dependent thalassaemia participated in the study

Table 2 reports on the profile of children with thalassemia. Most children were diagnosed with thalassaemia under the age of five (median age at diagnose: 1.95 years). The sex ratio of thalassaemic children was 1:1. Nearly 70% of them were diagnosed with Hb E-beta thalassaemia while 29.1% had beta-thalassaemia major. All thalassaemic children were transfusion-dependent and over 42% of them required blood transfusion at least twice a month. The current age of most thalassaemic children was below 20 years (Fig. 2).

**Table 2:** Profile of children with thalassaemia who required regular blood transfusion

Sex	n	%
Boy	184	50.4
Girl	181	49.6
Median age at diagnosis (years)	1.35 (N = 354)	
*Type of thalassaemia (N* = *361)*		
Beta thalassaemia	105	29.1
E-beta thalassaemia	253	70.1
Others (thalassaemia traits)	3	0.8
*The frequency of blood transfusion per month*		
1	172	47.1
2	154	42.2
3	30	8.2
> 3	9	2.5

### General knowledge

Table 3 presents knowledge of parents about thalassaemia. Over two-thirds of the respondents (~ 68%) correctly answered that thalassaemia is a genetic disease, while most of the participants (91.5%) correctly responded that thalassaemia is a not contagious disease. Only half of the parents of thalassaemic children correctly answered that thalassaemia is a preventable disease. Approximately half (45.5%) respondents were not aware that a thalassaemia carrier is healthy like normal people (16.4% answered: No, while 29% answered: Don’t Know).

**Table 3:** General knowledge about thalassaemia (correct, incorrect and don’t know)

Question (correct answer)	Responses		
	Correct	Incorrect	Don’t know
1. Thalassaemia is a contagious disease (NO)	334 (91.5%)	5 (1.4%)	26 (7.1%)
2. Thalassaemia is a genetic disease (Yes)	247 (67.7%)	84 (23%)	34 (9.3%)
3. Thalassaemia could be transmitted through blood transfusion from a person with thalassaemia (No)	307 (84.1%)	2 (0.6%)	56 (15.3%)
4. Marriage between two carriers can lead to a child with thalassaemia major (Yes)	321 (87.9%)	6 (1.6%)	38 (10.4%)
5. Thalassaemia carriers are as healthy as normal people (Yes)	199 (54.5%)	60 (16.4)	106 (29%)
6. Thalassaemia is a preventable disease (Yes)	188 (51.5%)	82 (22.5%)	95 (26%)
7. Thalassaemia is a completely curable disease (No)	247 (67.7%)	50 (13.7%)	68 (18.6%)
8. Which part of the human body or organ is affected by Thalassaemia? (Blood or circulatory system)	193 (52.9%)	10 (2.7%)	162 (44.4%)
9. Anyone could be a thalassaemia carrier including you. (Yes)	279 (76.4)	34 (9.3%)	52 (14.2%)
10. Thalassaemia can be identified by blood test (Yes)	303 (83%)	19 (5.2%)	43 (11.8%)

Table 4 reports the association between socio-demographic variables and level of knowledge about thalassaemia among caregivers. Mean knowledge scores were significantly higher in respondents with higher income and education. There were no differences in knowledge score in terms of gender, having thalassaemic children or a history of deaths related to thalassaemia in the extended family.

**Table 4:** Association between demographic variables and total thalassaemia knowledge scores

Variables	Mean score (SD) Highest score possible 10	Kruskal–Wallis *p* value
*Caregiver*		
Mother (n = 212)	7.30 (± 2.46)	0.974
Father (n = 153)	7.22 (± 2.616)	
*Monthly income*		
< 25 K(n = 225)	6.40 (± 2.509)	< 0.001
> 25 K(n = 140)	8.67 (± 1.821)	
*Fathers’ education*		
< 12 years(n = 183)	6.14 (± 2.483)	< 0.001
> 12 years(n = 182)	8.40 (± 2.008)	
*Mothers’ education*		
< 12 years (n = 226)	6.42 (± 2.499)	< 0.001
> 12 years (n = 139)	8.65 (± 1.868)	
*Thalassaemia patient in extended family*		
Yes (n = 80)	7.58 (± 2.151)	0.418
No (n = 285)	7.18 (± 2.615)	
*Death due to thalassaemia in extended family*		
Yes (n = 82)	6.99 (± 2.517)	0.225
No (n = 283)	7.35 (± 2.524)	

### Perceptions

Most respondents (~ 91%) had a guilty feeling for not undergoing premarital screening and most of them said they would have avoided marriage if they were well-informed about the sufferings of thalassaemia patients. Over two-thirds (~ 73%) of the parents were not planning another pregnancy. Approximately 95% of the parents wanted to connect with other thalassaemia families to share their experiences (Table 5).

**Table 5:** General perceptions of parents regarding thalassemia

Question	Responses		
	Yes	No	Not sure
Regretted not undergoing premarital screening	333 (91.2%)	32 (8.8%)	–
Interested in having further children	72 (19.7%)	270 (74%)	23 (6.3%)
Would you have married if you were informed about thalassemia?	12 (3.3%)	353 (96.7%)	–
Want to share personal experience with other parents	345 (94.5%)	20 (5.5%)	–

### Prenatal diagnosis

**Fig. 3:**
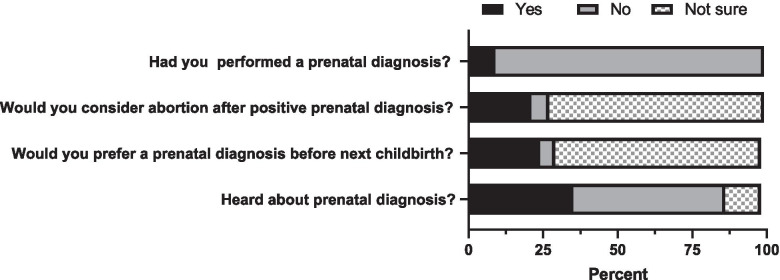
Knowledge, attitude, and practice for prenatal diagnosis in Bangladesh

Over one third of respondents (35.6%, n = 130) had heard about prenatal diagnosis. One fourth of parents (24.9%) would consider prenatal diagnosis in a future pregnancy, while 70% of them were unsure but interestingly only ~ 5% would decline prenatal diagnosis in a future pregnancy. On the other hand, nearly 21% parents would consider abortion in case of a positive prenatal test, 72.4% were unsure and only 6.6% of them would decline abortion. Only 9.3% (n = 34) mothers had a prenatal diagnosis in a previous pregnancy (Fig. 3) and three of those had terminated a pregnancy following a positive prenatal test.

### Barriers

**Fig. 4:**
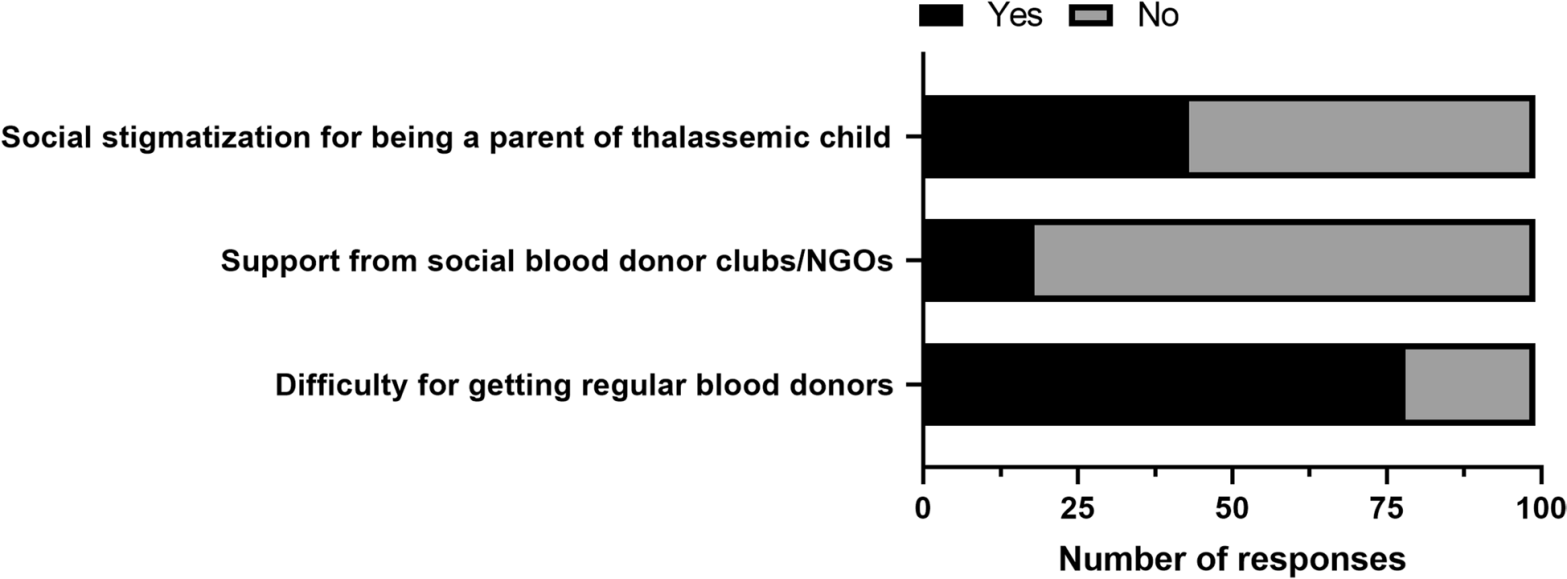
Barriers faced by parents of thalassemic children

Nearly 80% of the parents faced difficulty for obtaining blood donors regularly and a similar proportion (~ 81%) of them did not receive support from any organized blood clubs. More than 40% of the parents reported that they felt socially stigmatized (Fig. 4).

## Discussion

As far as we are aware, this was the first comprehensive study aimed to understand the parental perspective of knowledge gaps and perceptions regarding thalassemia, the barriers confronted by the parents for caring for their thalassaemic children in Bangladesh and their attitude to prenatal screening and prenatal diagnosis. Parents from all over Bangladesh (48 out of 64 administrative districts) participated in this study. Despite being caregivers of children with thalassemia, they had insufficient basic knowledge of thalassaemia and two-thirds of them had not heard about prenatal screening. Most parents reported that they were facing challenges in collecting blood for regular transfusion and a significant proportion of the parents felt social stigmatization.

### Poor general knowledge and regret

We found that almost all parents had not heard about the term ‘thalassemia’ before diagnosis of this disease in their children. This finding is consistent with our prior study involving college students (premarital age group) where over 65% respondents were unfamiliar with thalassaemia despite being the most common inherited condition in Bangladesh [13]. All these results indicate a significant lack of thalassaemia awareness in Bangladesh. Most parents would, in retrospect, have agreed to premarital screening if it were available. We also found that almost all parents would have avoided marriage if they had been aware of their own and their partner’s carrier status. In addition, almost all parents felt guilty for not undergoing pre-marital screening. All these findings suggest that thalassaemia is highly unfamiliar in the society and therefore the issue of awareness on thalassaemia should the cornerstone of future prevention strategy in Bangladesh. Besides, almost all thalassaemic children in our study were below 30 years of age. This essentially reflects the poor survival of thalassaemic children in Bangladesh, mainly due to inadequate treatment facilities and high out of pocket cost involved. This again underscore the importance of prevention of thalassaemic birth in a resource limited country like Bangladesh.

Most parents had knowledge about inheritance, diagnosis, carrier status, and the consequence of marriage between two carriers. These findings suggested better levels of knowledge compared to studies conducted in other South Asian countries [14–16]. However, nearly half of parents were not aware that carriers are usually healthy normal people without symptoms. This is particularly important since people may conceal their carrier status because of wrong perception. Higher family income and level of education were associated with higher knowledge scores.

### Prenatal diagnosis

Where both parents are known to be carriers of thalassaemia, prenatal diagnosis followed by therapeutic abortion has been part of effective thalassaemia prevention programmes in some countries [2]. However, our study has revealed that most parents (~ 65%) were not aware of prenatal diagnosis. our study showed that only 5.5% of parents were sure they would decline prenatal diagnosis and the remainder would go ahead with prenatal diagnosis and a large proportion (~ 70%) of them were unsure if they would accept prenatal diagnosis if offered in a future pregnancy. A small proportion (~ 9%) of parents opted for prenatal screening and there were three reported cases of therapeutic abortion. Another prior study reported 5% parents (n = 200) with thalassaemia children underwent prenatal diagnosis [17]. This extremely low level of awareness and practice of prenatal screening could be associated with the conservative religious culture in Bangladesh where over 90% people have Muslim faith. However, it is also very likely associated with the lack of knowledge of prenatal diagnosis, lack of resources and the fact that it is not offered free of charge.

Termination of pregnancy (TOP) for foetuses diagnosed with an abnormality (such as thalassemia) is a highly sensitive and debatable issue in Muslim countries around the world because TOP is not explicitly addressed by the Islamic scripture (Quran and Hadith) [18, 19]. However, a few Muslim countries permit TOP based on the interpretations (named as Fatwa) of Quran and Hadiths issued by respected religious scholars [18, 19]. A Fatwa is a non-binding Islamic legal opinion providing practical guidance for decision-making. In addition, a Fatwa is not country-specific and therefore, it could be utilized by Muslim anywhere in the world. Most importantly, Fatwas related to TOP are not available in public and these have been developed for health care practitioners (HCPs). Pakistan, a South Asian conservative Muslim country, introduced prenatal diagnosis (PND) for beta-thalassaemia in 1994 based on a Fatwa given by two respected Islamic Scholars. However, there is yet no national policy or law governing PND services [19] in Pakistan, although the province of Punjab has mandated premarital screening [20, 21]. Because of the unsettling situation, HCPs encounter dilemmas on religious and legal grounds [19]. Apart from unsolved legal and ethical issues, high cost and lack of awareness, the utilization of PND service is limited in Pakistan even though it has been available for over two decades [19]. In most Muslim countries, religious scholars may not be aware of medical conditions where religious opinion might be necessary to address certain emerging health problems. Therefore, engaging religious scholars in culturally sensitive health issues is expected to bring better policy outcomes.

In Bangladesh, three centres provide PND services, but to the best of our knowledge, there has yet to be a discussion with Bangladeshi religious scholars about this issue. Considering socio-cultural factors and poor awareness of thalassaemia among the general population, we suggest the current focus for the preventative strategy should be to encourage pre-marital screening (targeting high school or university students), so that prospective partners can make an informed decision about marriage which would entail a risk of thalassaemia in their offspring. At the same time, a national dialogue could be initiated on the prenatal diagnosis with relevant stakeholders including religious scholars.

### Barriers for blood transfusion and social acceptance

Regular blood transfusion is a fundamental aspect of treating thalassaemia. All thalassaemic children enrolled in our study were transfusion-dependent, requiring one to four bags of blood every month. Most families (~ 80%) reported they faced difficulty in collecting blood due to lack of support from organized donor’s clubs or NGOs. This situation is arguably worse at the community level. In Bangladesh, there is an acute crisis of blood supply due to lack of awareness for voluntary blood donation. Only 31% of estimated blood demand is met by voluntary blood donors [22]. Importantly, an unusual situation like the COVID-19 pandemic has put tremendous strain on blood supply and made the lives of thalassaemic children unbearable in South Asia including Bangladesh [23, 24]. Apart from on-demand blood transfusion, most thalassaemia patients in Bangladesh cannot afford conservative treatment because of resource-related and financial constraints. In our study, most children (~ 85%) attending the specialized centre were under 5 years of age suggesting a prevalence of premature death due to a lack of proper treatment.

In our study, nearly 40% parents experienced social stigmatisation, which could be associated with the nature of the disease. Blood is considered sacred in South Asian cultures, and this is often linked with identity and kinship. Therefore, any problem with blood could be attributed to corrupting blood [25]. An interrogative review of qualitative studies has shown that misperceptions regarding thalassaemia within a community could lead to social isolation of parents and their children; being labelled as criminals or God’s punished for sin. In-depth qualitative studies are warranted to understand the social stigmatisation in Bangladesh for devising mitigative interventions at community level [26].

## Limitations

There are some limitations in the present study. Firstly, the perspectives of parents who could not afford treatment in a thalassaemia specialized centre are not reflected in this study. Secondly, because of a convenience sampling strategy, study findings may not be generalisable, particularly to those who reside in large cities. In this study, parents from the majority of administrative districts of Bangladesh were included and therefore it is expected to represent the actual scenario as most patients reside in those areas. However, our study results would serve as a baseline for future studies.. The questionnaires have not been fully validated.

## Implications of this study

The current study illustrates a substantial knowledge gap among parents of thalassaemic children, their late realisation about the potentially preventable nature of thalassaemia, and their difficulties while caring for their children. In addition, this study noted poor transfusion services and shortage of blood supply chains, especially in rural areas of Bangladesh. These findings should be an eye-opener for the policymakers involved in national thalassemia prevention and treatment programs. A revision of existing national blood policies is required with the introduction of novel, setting-specific solutions to ensure adequate and timely supplies of safe blood and blood products for all patients. Mass awareness and educational programs are needed to alleviate the social stigma of thalassemia patients. Government and other stakeholders must join hands to ensure better survival and improve the quality of life of thalassaemia patients. As this is the first comprehensive study that tried to address the parental perspective of thalassaemia patients in Bangladesh, more research is necessary to refine and further elaborate these novel findings. In addition, this study underscores the need for research on other chronic diseases at the community level to identify shortcomings in the national health policies and find solutions to mitigate them. For clinicians and other personnel involved in the care of thalassaemia patients, this study serves as a reminder that parents of thalassaemic children need detailed counselling about the disease and its prognosis.

## Conclusion

This study has contributed information on attitude and practice about prenatal screening and prenatal diagnosis in Bangladesh. We found that most parents were not familiar with thalassaemia before diagnosis in their children and said they would have avoided marriage if they were well-informed. This study has also revealed the parental perspectives of regret, social stigmatisation, and inadequate support for managing regular blood transfusion. These findings would contribute to thalassaemia prevention and intervention strategies in Bangladesh as well as other similar settings elsewhere in the world.

## Data Availability

Data generated in this study are included in this article.
